# *ImputAccur:* fast and user-friendly calculation of genotype-imputation accuracy-measures

**DOI:** 10.1186/s12859-022-04863-z

**Published:** 2022-08-04

**Authors:** Kolja A. Thormann, Viola Tozzi, Paula Starke, Heike Bickeböller, Marcus Baum, Albert Rosenberger

**Affiliations:** 1 Institute of Computer Science, Georg-August-University Göttingen, 37077 Göttingen, Germany; 2 Department of Genetic Epidemiology, University Medical Center Göttingen, 37079 Göttingen, Germany

**Keywords:** Imputation, Accuracy, GWAS, Marker selection, SNP, Quality control

## Abstract

**Background:**

*ImputAccur* is a software tool to measure genotype-imputation accuracy. Imputation of untyped markers is a standard approach in genome-wide association studies to close the gap between directly genotyped and other known DNA variants. However, high accuracy for imputed genotypes is fundamental. Several accuracy measures have been proposed, but unfortunately, they are implemented on different platforms, which is impractical.

**Results:**

With *ImputAccur,* the accuracy measures *info, Iam-hiQ* and *r*^*2*^*-based* indices can be derived from standard output files of imputation software. Sample/probe and marker filtering is possible. This allows e.g. accurate marker filtering ahead of data analysis.

**Conclusions:**

The source code (Python version 3.9.4), a standalone executive file, and example data for *ImputAccur* are freely available at https://gitlab.gwdg.de/kolja.thormann1/imputationquality.git.

**Supplementary Information:**

The online version contains supplementary material available at 10.1186/s12859-022-04863-z.

## Background

Commercial single nucleotide polymorphism (SNP) microarrays are used to genotype DNA samples for genome-wide association studies (GWAS). Usually, between 300,000 and 4 million variants are genotyped. Imputation methods have been developed to close the gap between genotyped and existing DNA variants [1–3]. Most methods estimate a posteriori genotype probabilities $p_{g,i,m}$ (one of three possible genotypes *g*) for each untyped SNP/variant/marker *m* and each individual *i* in the sample of interest. The resulting increased variant density improves the genomic coverage and may raise the power to detect associations with a trait [4]. Quality control of the imputation is essential, e.g. to exclude poorly imputed variants from statistical analysis. Several quality indices have been developed and are routinely applied in studies [2, 3, 5]. These comprise *inter alia* MACH’s *r*^2^, BEAGLES’s *r*^2^, IMPUTE2’s *info* or the recently proposed *Iam hiQ,* including a regional classification across markers [6]. Unfortunately, these accuracy measurements are implemented on different platforms. With *ImputAccur,* the comfortable use of all these indices is possible.

Furthermore, *ImputAccur* classifies markers to be located in a “cold”, “tepid”, “hot”, or “very hot” region, the last indicating massively inaccurate imputation, as outlined by Rosenberger et al. [6] Details and equations of these accuracy indices and the classification are summarized in the Additional file 1. The validity of the calculations was tested by comparison with output files from IMPUTE2 (for info) and with known results of carefully selected sample data (all indices).

## Implementation

*ImputAccur* requires the user to provide marker information (leading information) along with the estimated a-posteriori genotype probabilities (dosages) as an input file, which is a plain text file (or zipped). These are standard files generated by imputation software. Each row contains information on one marker. The second and third columns should contain [2] a unique marker name and [3] its physical position (e.g. on the chromosome). Probabilities for the genotypes 0, 1, and 2 of each sample/individual can be contained in 3 (summing to 1) or 2 (amended to 1) columns. Missing or inaccurate imputations are indicated by negative values. Hence, the number (no.) of rows in the input file equals the no. of genomic markers; the no. of columns equals the no. of leading columns + 2/3 times the no. of samples/individuals.

Basic settings for program control (e.g. name and path of the input file) and/or the structure of the input files (e.g. number of leading columns, 2/3 genotype probabilities) can be defined in an additional parameter file (params.txt). There is also the option to specify files containing either markers (matching to marker names) or samples/individuals (numbers matching to column order in the input-file) to be excluded from the calculation. One can also provide names for the leading columns; however, the second and third column will always be named “SNP” and “position”.

### Launching the application

To invoke the Python code of *ImputAccur,* the user may use the following command syntax:

$${\text{runfile}}\left( {\text{`}}\left[ {{\text{NAME}}\;{\text{OF}}\;{\text{PROGRAM}}} \right].{\text{py}}{\text{'}},\;{\text{args}} = {\text{`}}{\text{ - f}}\;{\text{params}}.{\text{txt}}{\text{'}} \right).$$

Alternatively, one can run *ImputAccur* as an executable file (*ImputAccur.exe*) or without the parameter file (e.g. on a Windows operating system). The program will then ask for the parameters to be entered interactively. For use on Ubuntu, the program can be started via the terminal by navigating to the folder containing the program and parameter file and entering “python [NAME OF PROGRAM].py -f params.txt”. Alternatively, it can be started using only the command “python [NAME OF PROGRAM].py” in the corresponding folder. The program will then ask for the parameters one at a time as well.

### Runtime/performance

*ImputAccur* needed less than 0.8 s per marker to calculate the accuracy indices based on dosages of 10,000 probes/individuals. This was carried out on the High Performance Computing (HPC) clusterof the University of Göttingen/GWDG (https://www.gwdg.de/hpc). The calculation took less than 0.08 s for 1000 probes, less than 0.008 s for 100 probes, and so on.

We assessed the performance of *ImputAccur* on Scientific Linux and on Ubuntu 18.04.6 with Python 3.9.4, 3.7.3, and 3.6.13., as well as on Windows 10 Pro Build 21H2.

## Results/example

Assume your input-file (see example *test1* in the Additional file 1) contains information of 7 SNPs in three leading columns and 3 genotype probabilities each of 5 samples/individuals. Hence, the file has 7 rows and 3 + 3 × 5 = 18 columns. Because the SNPs rs00001, rs00003, and rs00004 are quality control markers, these are listed in the file *exclude_SNP.txt*. Because individuals 1 and 2 are external controls, these are listed in *exclude_PROBE.txt*.

This is the input file (*test1.imputed*):

**Table:** 

1 rs00001 913 1 0 0 1 0 0 1 0 0 1 0 0 1 0 0
2 rs00002 402 0.01 0.99 0 0.01 0.99 0 0.01 0.99 0 0.01 0.99 0 0.01 0.99 0
3 rs00003 644 0.333 0.334 0.333 0.333 0.334 0.333 0.333 0.334 0.333 0.333 0.334 0.333 0.333 0.334 0.333
4 rs00004 222 0.25 0.5 0.25 0.25 0.5 0.25 0.25 0.5 0.25 0.25 0.5 0.25 0.25 0.5 0.25
5 rs00005 221 0.47 0.18 0.35 0.89 0.02 0.09 0.03 0.96 0.01 0.94 0 0.06 0.62 0.34 0.04
6 rs00006 955 0.975 0.002 0.023 0.52 0.154 0.326 0.309 0.21 0.481 0.48 0.509 0.011 0.969 0.004 0.027
7 rs00007 518 0.63 0.14 0.23 0.86 0.09 0.05 0.35 0.24 0.41 0.01 0.23 0.76 0.76 0.05 0.19

For this, one needs to set the following program parameters in *params.txt* or during the execution:

**Table:** 

-i	test1.imputed	[path to and name of input-file]
-l	3	[number of leading columns]
-c	0	[third genotype probability needs to be calculated—TRUE (1) or FALSE (0)]
-p	excluded_PROBE.txt	[path to and name of sample exclusion file]
-m	excluded_SNP.txt	[path to and name of SNP exclusion file]
-n	SNP_no,SSSS,PPPPPP	[names for leading columns]

This is the output (*test1.accuracy*):

**Table:** 

SNP_no	SNP	Position	N	MAF (%)	Iam_chance_	Iam_HWE_	hiQ	Accuracy	Info	${\text{r}}_{{{\text{MACH}}}}^{2}$	${\text{r}}_{{{\text{Beagle}}}}^{2}$
5	rs00005	221	5	26.0	0.546	0.448	0.739	HOT	0.099	0.319	0.113
2	rs00002	402	5	49.5	0.97	0.968	0.929	TEPID	0.98	0.0	− 9
7	rs00007	518	5	40.3	0.344	0.289	0.72	HOT	− 0.057	0.631	0.294
6	rs00006	955	5	26.1	0.446	0.326	0.963	HOT	− 0.056	0.488	0.179

### Output interpretation (for marker rs00005)

A-posteriori genotype probabilities of N = 5 individuals were contained in the input file for SNP rs00005 (fifth in the input file) at position 221 on the considered chromosome. A frequency (MAF) of 26% for the minor allele can be derived from these dosages. According to the accuracy indices *Iam*_*chance*_ (0.546) and *Iam*_*HWE*_ (0.448) it is reasonable to assume that about half of the information contained in dosages comes from the true (but unknown) genotypes of the individuals in the sample, the other half comes from the population used as a reference for genotype imputation. The values are borderline near the recommended threshold of 0.47 [6]. The difference between *Iam*_*chance*_ and *Iam*_*HWE*_ is the “anchor point” used, which is either purely populations-related dosages (Hardy–Weinberg Equilibrium HWE, taking MAF into account) or pure chance (1/3 probability for each of the three possible genotypes of a SNP). *Iam*_*chance*_ and *Iam*_*HWE*_* usually have comparable* values [6].

A value of 0.099 for info indicates that only 10% of the statistical information on the minor population allele frequency (MAF), given “known” genotypes, remained after the genotypes had been imputed for rs00005 [3]. For the measure *info,* threshold values such as 0.8 or 0.3 have been proposed, but without sound justification [3, 7, 8].

*A* value of 0.739 for hiQ indicates insufficient *heterogeneity of dosages across all* samples/individuals, as it is lower than the recommended threshold of 0.97 [6]. The imputation seems to have resulted in dosages too similar to be used for statistical inference testing.

Since ${\text{r}}_{{{\text{MACH}}}}^{2}$ has a value of 0.319, one can conclude that the power of an allelic test, in the case of a binary trait, based on the imputed genotypes of rs00005 is approximately $\sqrt {0.319} = 0.56$ times that of the same test if all genotypes were present. The same applies for ${\text{r}}_{{{\text{Beagle}}}}^{2}$ [2].

Overall, all indices identify rs00005 as a marker with poor imputation. The “accuracy” is rated as “hot”, indicating that rs00005 is also located in a genomic region enriched with markers of poor imputation.

**Fig. 1:**
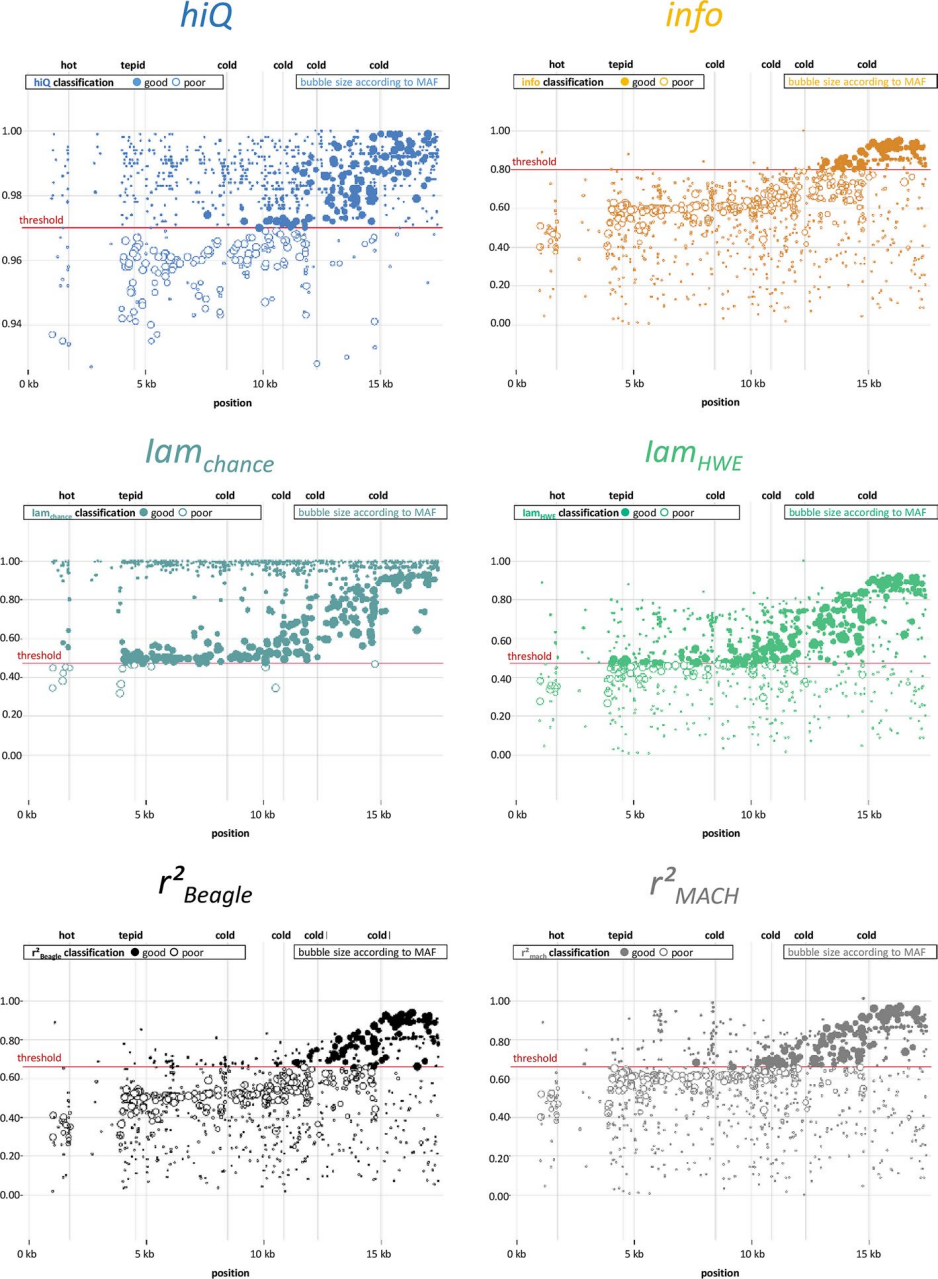
Example of real-data genotype-imputation accuracy-measures for the telomere region on chromosome 9. Top left: hiQ, top right: info, centre left: Iam_chance_, centre right: Iam_HWE_, bottom left: ${\text{r}}_{{{\text{Beagle}}}}^{2}$, bottom right: ${\text{r}}_{{{\text{MACH}}}}^{2}$; each dot represents one imputed marker; the marker size is according to minor allele frequency (MAF); threshold values are freely selectable; vertical lines: centre of region classified as: “cold”, “tepid”, “hot”, or “very hot” (the definition is given in the Additional file 1).

In addition, Fig. 1 illustrates the regional accuracy of all indices, including the classification from “cold” to “very hot”. The telomere region of 0 to 17.5 kb of chromosome 9 is plotted. One can easily see that the imputation is not accurate near the ends of each sister chromatid. One can also see the differences between the accuracy indices, especially for rare markers (low MAF—small dots), and realize how critical the choice of appropriate thresholds can be (including the classification implemented in *ImputAccur*). The example data used are described elsewhere [6].

## Conclusion

*ImputAccur* is an easy-to-use software to determine multiple measures of accuracy for imputed genotypes and independent on the imputation platform used. This allows greater flexibility in post-imputation variant filtering. Because it also delivers regional classification, poorly imputed chromosome segments may be identified.

## Availability and requirements

- Project name: *ImputAccur*
- Project home page: https://gitlab.gwdg.de/kolja.thormann1/imputationquality.git
- Operating system(s): Platform independent
- Programming language: Python version 3.9.4
- Other requirements: none
- License: The GitHub Terms of Service/2-Clause BSD License
- Any restrictions to use by non-academics: none

## Supplementary Information

**Additional file 1.** Equations for calculating the accuracy measures and the scheme for classifying genomic regions.

## Data Availability

The source code (Python version 3.9.4), a standalone executive file, and a user manual of *ImputAccur* are freely available at https://gitlab.gwdg.de/kolja.thormann1/imputationquality.git. Example data are available in the same repository. The example data presented in Fig. 1 are available from ILCCO/INTEGRAL but restrictions apply to the availability of these data, which were used under license for the current study, and so are not publicly available. Data are however available from the authors upon reasonable request and with permission of ILCCO/INTEGRAL.

## Abbreviations

GWAS: Genome-wide association studies
SNP: Single nucleotide polymorphism
MAF: Minor allele frequency
Dosages: Estimated a-posteriori genotype probabilities
HWE: Hardy–Weinberg equilibrium

## References

[1] Hickey JM, Cleveland MA, Maltecca C, Gorjanc G, Gredler B, Kranis A (2013). Genotype imputation to increase sample size in pedigreed populations. Methods Mol Biol.

[2] Das S, Abecasis GR, Browning BL (2018). Genotype imputation from large reference panels. Annu Rev Genom Hum Genet.

[3] Marchini J, Howie B (2010). Genotype imputation for genome-wide association studies. Nat Rev Genet.

[4] Winkler TW, Day FR, Croteau-Chonka DC, Wood AR, Locke AE, Magi R (2014). Quality control and conduct of genome-wide association meta-analyses. Nat Protoc.

[5] Browning BL, Browning SR (2009). A unified approach to genotype imputation and haplotype-phase inference for large data sets of trios and unrelated individuals. Am J Hum Genet.

[6] Rosenberger A, Tozzi V, Bickeböller H, Hung RJ, Christiani DC, Caporaso NE (2022). Iam hiQ—a novel pair of accuracy indices for imputed genotypes. BMC Bioinform.

[7] Mitt M, Kals M, Parn K, Gabriel SB, Lander ES, Palotie A (2017). Improved imputation accuracy of rare and low-frequency variants using population-specific high-coverage WGS-based imputation reference panel. Eur J Hum Genet.

[8] Krithika S, Valladares-Salgado A, Peralta J, Escobedo-de LaPena J, Kumate-Rodriguez J, Cruz M (2012). Evaluation of the imputation performance of the program IMPUTE in an admixed sample from Mexico City using several model designs. BMC Med Genom.

