# Supplementary material for: ImputAccur: fast and user-friendly calculation of genotype-imputation accuracy-measures
Source: BMC Bioinformatics. 2022 Aug 4;23:316. doi: 10.1186/s12859-022-04863-z (PMC9351229; doi:10.1186/s12859-022-04863-z)
Supplement: Supplementary file 1 — Additional file 1. Equations for calculating the accuracy measures and the scheme for classifying genomic regions. [file 12859_2022_4863_MOESM1_ESM.pdf]

Supplement to

ImputAccur: fast and user-friendly calculation of genotype-imputation accuracy-measures

Thormann A Kolja [1], Tozzi Viola [2], Starke Paula [2], Bickeböller Heike [2], Baum Marcus [1], Rosenberger Albert [2]

1. Institute of Computer Science , Georg-August-University Göttingen, 37077 Göttingen, Germany
2. Department of Genetic Epidemiology, University Medical Centre Göttingen, 37079 Göttingen, Germany

Accuracy measures

For a di-allelic SNP denote the genotypes *g* by 0, 1 or 2 according to the number of minor alleles A, with minor allele frequency (MAF) f_A in the population. A-posteriori genotype probabilities $p_{g,i,m}$ are given for each untyped SNP/marker *m* and each individual *i*. The *expected* allele dosage of ith probe/individual for the mth SNP/marker is given as $e_{im} = p_{1im} + 2p_{2im}$. We also define $f_{im} = p_{1im} + 4p_{2im}$. To avoid incomputable indices, MAF is estimated as $\widehat{f_A} = \frac{1+\sum_{i=1}^{N} e_{im}}{2+2N}$, with N being the number of samples/individuals, but not less than 1000. The genotypes are in Hardy-Weinberg Equilibrium (HWE) if $[p_0 \quad p_1 \quad p_2] = [f_A^2 \quad 2f_A(1-f_A) \quad (1-f_A)^2]$.

IMPUTE2's *info,* is defined as $$info_m = 1 - \frac{\sum_{i=1}^{N}(f_{im} - e_{im}^2)}{2N\hat{f}_A(1-\hat{f}_A)}.$$

It can be regarded as the proportion of statistical information on MAF in the imputed genotypes, relative to “known” genotypes (1 Supplementary Information S3).

MACH $\hat{r}^2$ is defined as $$\hat{r}_m^2 = \left[\frac{\sum_{i=1}^{N} e_{im}^2}{N} - \left(\frac{\sum_{i=1}^{N} e_{im}}{N}\right)^2\right] / 2\hat{f}_A(1-\hat{f}_A).$$

It can be regarded as ratio of the empirically to the expected variance of the allele dosage (under HWE) (1 Supplementary Information S3).

BEAGLE R² is defined as: $$R_m^2 = \frac{\left[\sum_{i=1}^{N} z_{im}e_{im} - \frac{1}{N}\left(\sum_{i=1}^{N} z_{im} \sum_{i=1}^{N} e_{im}\right)\right]^2}{\left[\sum_{i=1}^{N} f_{im} - \frac{1}{N}\left(\sum_{i=1}^{N} e_{im}\right)^2\right]\left[\sum_{i=1}^{N} z_{im}^2 - \frac{1}{N}\left(\sum_{i=1}^{N} z_{im}\right)^2\right]},$$

with $z_{im} \in \{0, 1, 2\}$ being the most likely imputed genotype *g*. It is the correlation of the best-guess genotype and the allele dosage (1 Supplementary Information S3).

Iam is a rescaled measure of anti-concentration index $Q_{i,m} = \sum_{g=1}^{3} p_{g,i,m}(1-p_{g,i,m})$, or averages over all marker as $\bar{Q}_m$ (2). Because $Q_{i,m}$ can take values between 0 and 2/3 (in the case of equally likely genotypes: $p_{g,i,m}=1/3$),

Iam$_{chance}$ is defined as $$Iam_{chance,m} = 1 - \frac{\bar{Q}_m}{2/3}.$$

Considering the genotype probabilities in HWE as natural reference point,

Iam$_{HWE}$ is defined as $$Iam_{HWE,m} = 1 - \frac{\bar{Q}_m}{Q_{HWE,m}}.$$

r^2-based measures and *info* are directly related to the power of the statistical test of a marker-trait association. Marchini et al. (1) showed, that *info*, r^2_{MACH} and r^2_{BEAGLE} correlate strongly, but can also exceed 1 or be undefined. In contrast, Rosenberger et al. (2) showed that *Iam hiQ* and *info* carry different information on imputation accuracy and complement each other as indices.

Implementation

ImputAccur is a software tool to calculate the genotype-imputation accuracy-measures Iam_{chance}, Iam_{HWE}, *hiQ, info*, r^2_{MACH} *and* r^2_{BEAGLE}, independent of the imputation methods applied. All that is needed are dosage files. In contrast to the platform-depending solutions, entire samples/individuals or SNPs can be excluded (or explicitly included) from the calculation. With this, e.g. control samples and control markers or non-study persons can be treated adequately.

Classification of genomic regions

Furthermore, *ImputAccur* classifies markers to be located in a “cold”, “tepid”, “hot” or “very hot” region, the last indicating massive inaccurate imputation, as outlined by Rosenberger et al. (2) Therefore, the exponentially weighted moving average (EWMA) is used to determine the average of the imputation accuracy (by Iam_{HWE} *hiQ*) of markers around the considered SNP. Based on this, genomic regions are categorized from "cold" to "very hot", the latter indicating massively inaccurate imputation.

S-Table 1: accuracy classification scheme

Iam$_{HWE}$		hiQ	accuracy
NA	or	NA	"NA"
≥ 0.47	and	≥ 0.97	"cold"
< 0.47	or	< 0.97	"tepid"
< 0.47	and	< 0.97	"hot"
$< 0.47/2$	and	0.97/2	"very hot"

NA (Not Available) missing value;
thresholds according to Rosenberger et al. (2)

References

1. Marchini J, Howie B. Genotype imputation for genome-wide association studies. Nat Rev Genet. Juli 2010;11(7):499–511.

2. Rosenberger A, Tozzi V, Bickeböller H, Hung RJ, Christiani DC, Caporaso NE, u. a. Iam hiQ—a novel pair of accuracy indices for imputed genotypes. BMC Bioinformatics. 24. Januar 2022;23(1):50.
